# Optimization of magnetic resonance imaging protocol for the diagnosis of transient global amnesia

**DOI:** 10.1590/0100-3984.2018.0028

**Published:** 2019

**Authors:** Luiz de Abreu Junior, Laiz Laura de Godoy, Luciana Pinheiro dos Santos Vaz, André Evangelista Torres, Angela Maria Borri Wolosker, Ulysses Santos Torres, Maria Lucia Borri

**Affiliations:** 1 Department of Neuroradiology, Grupo Fleury, São Paulo, SP, Brazil.; 2 Hospital das Clínicas da Faculdade de Medicina da Universidade de São Paulo (HC-FMUSP), São Paulo, SP, Brazil.; 3 Hospital São Paulo, Escola Paulista de Medicina da Universidade Federal de São Paulo (EPM-Unifesp), São Paulo, SP, Brazil.

**Keywords:** Amnesia, Amnesia, transient global, Memory, Hippocampus, Magnetic resonance imaging, Diffusion magnetic resonance imaging, Amnésia, Amnésia global transitória, Memória, Hipocampo, Ressonância magnética, Difusão

## Abstract

**Objective:**

To emphasize the most appropriate magnetic resonance imaging (MRI) diffusion protocol for the detection of lesions that cause transient global amnesia, in order to perform an accurate examination, as well as to determine the ideal time point after the onset of symptoms to perform the examination.

**Materials and Methods:**

We evaluated five patients with a diagnosis of transient global amnesia treated between 2012 and 2015. We analyzed demographic characteristics, clinical data, symptom onset, diffusion techniques, and radiological findings. Examination techniques included a standard diffusion sequence (b value = 1000 s/mm^2^; slice thickness = 5 mm) and a optimized diffusion sequence (b value = 2000 s/mm^2^; slice thickness = 3 mm).

**Results:**

Brain MRI was performed at 24 h or 36 h after symptom onset, except in one patient, in whom it was performed at 12 h after (at which point no changes were seen) and repeated at 36 h after symptom onset (at which point it showed alterations in the right hippocampus). The standard and optimized diffusion sequences were both able to demonstrate focal changes in the hippocampi in all of the patients but one, in whom the changes were demonstrated only in the optimized sequence.

**Conclusion:**

MRI can confirm a clinical hypothesis of transient global amnesia. Knowledge of the optimal diffusion parameters and the ideal timing of diffusion-weighted imaging (> 24 h after symptom onset) are essential to improving diagnostic efficiency.

## Abstract (pt)

**Objetivo:**

Enfatizar o protocolo de difusão mais adequado para detecção de lesões da amnésia global transitória, a fim de realizar um exame preciso, em tempo ideal, após o início dos sintomas.

**Materiais e Métodos:**

Foram analisados cinco pacientes com diagnóstico de amnésia global transitória atendidos entre 2012 e 2015, considerando-se dados demográficos, clínicos, tempo do início dos sintomas, técnicas de difusão e achados radiológicos. As técnicas incluíram uma sequência de difusão padrão (b = 1000 s/mm^2^; espessura do corte = 5 mm) e uma sequência de difusão otimizada (b = 2000 s/mm^2^; espessura de corte = 3 mm).

**Resultados:**

A ressonância magnética de encéfalo foi realizada após 24 ou 36 horas do início dos sintomas, exceto em um paciente, em que foi realizada após 12 horas (sem alterações) e repetida após 36 horas (mostrando alterações hipocampais). Em todos os pacientes foram demonstradas alterações focais na difusão no hipocampo em ambas as técnicas, exceto em um paciente, em que as alterações foram demonstradas apenas na sequência otimizada.

**Conclusão:**

A ressonância magnética é capaz de confirmar a hipótese clínica de amnésia global transitória. O conhecimento dos parâmetros ótimos da técnica de difusão e o melhor tempo para a detecção das alterações (> 24 horas) são essenciais para aprimorar a eficiência diagnóstica.

## INTRODUCTION

Transient global amnesia (TGA) is a syndrome characterized by the sudden onset of anterograde amnesia, with or without a retrograde component, lasting up to 24 h, without affecting other neurological functions and without long-term sequelae^(^^1^^)^.

The clinical characteristics of TGA have been well described, although the etiology and pathophysiology of the condition remain obscure. The memory impairment suggests that the disease affects the structures within the temporal lobe^(^^2^^)^. Structural magnetic resonance imaging (MRI) is capable of detecting abnormalities in the structures of the memory circuits in the mesiotemporal region^(^^3^^)^.

Although the altered diffusion in TGA resembles that seen in the temporal evolution of ischemic lesions, sequelae affecting the hippocampal structures and detectable on MRI have yet to be observed in follow-up studies^(^^2^^)^.

High-resolution MRI shows aspects characteristic of TGA and can facilitate its diagnosis. The neuroimaging findings in TGA also suggest involvement of the memory circuits in the mesiotemporal region, where focal lesions with hyperintense signals can be seen on T2-weighted images, together with restricted diffusion in the lateral hippocampus on diffusion-weighted imaging (DWI). There is a selective distribution in the cornu ammonis 1 region of the hippocampus, suggesting selective vulnerability of these neurons to metabolic stress^(^^4^^)^. In patients with TGA, the focal lesions, which vary in size from 1 mm to 5 mm, can be single or multiple and can be unilateral or bilateral^(^^5^^)^. Such lesions can also present as edema-like hyperintense foci on T2-weighted images^(^^3^^)^.

The rate of detection of focal lesions in TGA is directly dependent on their temporal evolution and on optimization of the MRI parameters^(^^6^^)^. On DWI, such lesions are usually identified in the first 24-72 h and can typically still be detected until 7-10 days after symptom onset^(^^4^^)^. The maximum level of detection occurs within 48-72 h after the onset of symptoms. Images obtained within the first 24 h might not show such lesions; therefore, if no lesion is detected in the initial DWI study, especially if it is performed within a few hours after symptom onset, it is recommended that a follow-up DWI study be performed a few days thereafter^(^^6^^)^. As for optimization of the MRI parameters, studies in the literature suggest that the combined use of a higher b value (> 2000 s/mm^2^) and thinner slices (< 3 mm) is ideal for the detection of TGA^(^^6^^)^.

Imaging facilities that have a more generalist profile or treat neurological patients only sporadically might not have an optimized protocol for the study of TGA, using standard sequences that do not include ideal parameters for the characterization of the subtle findings associated with the condition. It should also be borne in mind that the use of higher b values in the DWI sequences can impair the spatial resolution. In view of those aspects, together with the fact that there are as yet no studies validating this optimized technique in the radiology literature of Brazil, we proposed to evaluate the effects of the use of this protocol in a series of cases.

## MATERIALS AND METHODS

This was a retrospective study evaluating patients with TGA seen at our facility between January 2012 and December 2015. In all of the patients, the diagnosis of TGA had been confirmed on the basis of the clinical findings.

The clinical diagnosis of TGA was based on the criteria established by Caplan and Hodges^(^^7^^,^^8^^)^: anterograde amnesia, witnessed by an observer; no clouding of consciousness or loss of personal identity; cognitive impairment being limited to the amnesia; no focal neurological signs or epilepsy; no recent history of head injury or seizures; and resolution of symptoms within 24 h.

The brain MRI of the patients included in the study produced characteristic imaging findings-focal lesions, measuring 1-5 mm, with hyperintense signals on T2-weighted images and restricted diffusion in the lateral hippocampus (all lesions were selectively detected in the cornu ammonis 1 region of the hippocampus)-which corroborated the diagnosis. All of the DWI examinations were performed in an MRI scanner (Gyroscan Intera; Philips Medical Systems, Best, The Netherlands), operating at 1.5 T. The study technique included a standard diffusion sequence (b value = 1000 s/mm^2^; slice thickness = 5 mm) and an optimized diffusion sequence (b value = 2000 s/mm^2^; slice thickness = 3 mm). All of the images were evaluated by a radiologist with 21 years of experience.

The study sample included five patients (three women and two men), with a mean age of 65.3 years, In all of the patients, we evaluated the following parameters: the approximate duration of the TGA episode; the time from symptom onset to brain MRI; the characteristics of the focal lesions (single or multiple and unilateral or bilateral) showing restricted diffusion in the hippocampus; an association with a Valsalva maneuver (e.g., severe cough and difficult evacuation); a history of clinically similar episodes (recurrence?); and the visualization of the lesion according to the study technique.

In the physical examination, all of the patients were lucid. They were oriented in time and space, with no motor, sensory, gait, visual or other associated neurological symptoms. None of them showed any significant biochemical abnormalities.

## RESULTS

The demographic and clinical characteristics of the patients, as well as the timing, technique, and findings of brain MRI, are summarized in Table 1. The mean age of the five patients evaluated was 65.3 years (range, 59-71 years): the three women were 59, 64, and 69 years old, respectively; and the two men were 63 and 71 years old, respectively. All five patients presented to the emergency department with current or previous sudden-onset amnesia lasting an average of 7 h (range, 4-12 h), without impairment of any other neurological functions. Two of the patients reported having inadvertently performed a Valsalva maneuver shortly before the onset of the TGA episode (one reporting severe coughing and the other reporting difficult evacuation). One of the patients reported having experienced a similar episode approximately six months prior, indicative of recurrent TGA.

**Table 1:** Epidemiological, clinical and brain MRI findings of five patients diagnosed with TGA.

Variable	Patient 1	Patient 2	Patient 3	Patient 4	Patient 5
Demographic characteristics					
Age	64 years	71 years	69 years	63 years	59 years
Gender	Female	Male	Female	Male	Female
Estimated duration of the TGA episode	9 h	4 h	12 h	4 h	6 h
Association with of a Valsalva maneuver (intense cough, difficult evacuation)	-	-	Intense cough preceding the symptoms	-	Difficult evacuation preceding the symptoms
History of clinically similar episodes	-	-	-	-	Similar episode six months prior
Brain MRI					
Approximate time from symptom onset to MRI	36 h	24 h	24 h	12 h: normal; 36 h: altered	36 h
Characteristics of the focal lesions with restricted diffusion in the hippocampus	Two (bilateral) hippocampal foci (tail on the right and body on the left)	Single hippocampal focus (body on the left)	Two (bilateral) hippocampal foci (both in the body)	Single hippocampal focus (head on the right)	Single hippocampal focus (body on the left)
DWI sequence employed					
Standard sequence: b = 1000 s/ mm^2^; slice thickness = 5,0 mm	Lesion(s) visualized	Lesion(s) visualized	Lesion(s) not visual-ized	Lesion(s) visualized	Lesion(s) visualized
Additional sequence: b = 2000 s/ mm^2^; slice thickness = 3,0 mm	Lesion(s) visualized	Lesion(s) visualized	Lesion(s) visualized	Lesion(s) visualized	Lesion(s) visualized

**Figure 1:**
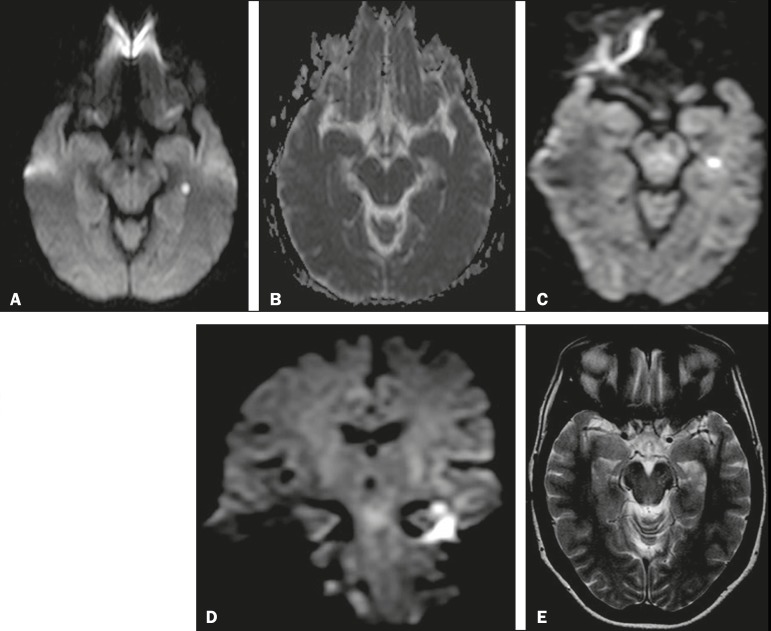
A focus of marked restricted diffusion in the lateral portion of the body of the left hippocampus, measuring approximately 5 mm. **A:** DWI sequence with a b value of 1000 s/mm^2^ and a slice thickness of 5 mm. **B:** Apparent diffusion coefficient map. **C:** DWI sequence with a b value of 2000 s/mm^2^ and a slice thickness of 3 mm. **D:** Coronal DWI sequence of the hippocampus with a b value of 2000 s/mm^2^ and a slice thickness of 3 mm. **E:** T2-weighted sequence showing an edema-like focus with a hyperintense signal.

**Figure 2:**
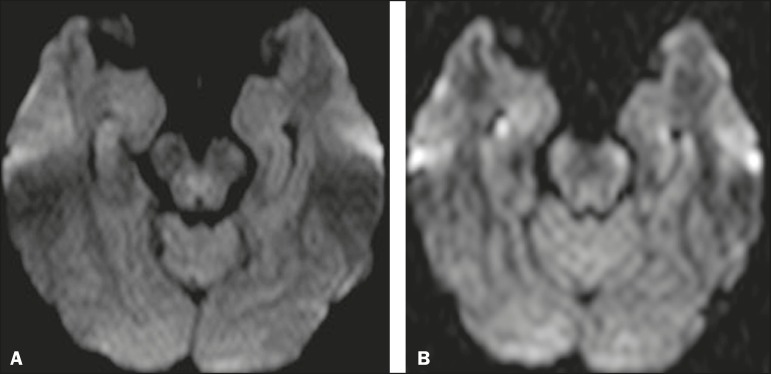
**A:** DWI sequence with a b value of 1000 s/mm^2^ and a slice thickness of 5 mm, showing no abnormalities. **B:** DWI sequence with a b value of 2000 s/mm^2^ and a slice thickness of 3 mm, showing tiny foci with hyperintense signals in both hippocampal bodies, more evident on the right, in the same patient.

In all but one of the patients with suspected TGA evaluated in the present study, brain MRI was performed at 24 h or 36 h after symptom onset. In the remaining patient, it was initially performed 12 h after symptom onset, at which point it showed no abnormalities. In that same patient, a second brain MRI, performed at 36 h after symptom onset, showed characteristic hippocampal changes. Three (60%) of the patients presented a single focus of restricted diffusion in the hippocampus (Figure 1): in the head of the right hippocampus in one; and in the body of the left hippocampus in two. The other two patients (40%) each presented two foci of restricted diffusion in the hippocampus, the foci being bilateral in both (Figure 2): in the tail of the right hippocampus and body of the left hippocampus in one; and in both hippocampal bodies in the other.

In the present study, the standard diffusion sequence was performed initially, an optimized diffusion sequence then being performed when TGA was suspected. In four (80%) of the five patients, both diffusion sequences showed focal changes in the hippocampus, whereas such changes were detected only in the optimized sequence in the one remaining patient (Figure 2).

## DISCUSSION

TGA predominantly affects patients in the age group of 50-70 years. The reported annual incidence of the condition is 3.4-10.4 cases/100,000 population. In the population over 50 years of age, its incidence is 23.5 cases/100,000 population^(^^1^^)^. It is more common in individuals with migraine, especially in female patients 40-60 years of age^(^^9^^)^, as was also observed in our study sample. The annual recurrence rate of TGA is between 6% and 10%^(^^10^^)^. In the present study, only one patient reported a previous TGA episode (six months prior, indicative of recurrent TGA).

Some events are often reported as preceding a TGA episode, including sudden immersion in cold or hot water; physical exertion; emotional or psychological stress; pain; medical procedures; sexual intercourse; and Valsalva maneuvers. Such events have been implicated in 50-90% of documented TGA episodes^(^^3^^)^. Two of the five patients evaluated in the present study reported having inadvertently performed a Valsalva maneuver shortly before the onset of the TGA episode (during severe coughing in one and during difficult evacuation in the other).

As previously mentioned, the diagnosis of TGA is made on the basis of the clinical findings and can be confirmed by the MRI findings^(^^11^^)^.

Although not yet consistently proven, some hypotheses have been proposed regarding the pathophysiology of TGA, including venous flow abnormalities, focal ischemia, migraine, and epileptic phenomena^(^^4^^)^. Given the abrupt onset of symptoms and restricted diffusion seen in TGA, thromboembolic mechanisms have been suggested; however, several studies have failed to show an association between TGA and cardiovascular risk factors, such as systemic arterial hypertension and hypercholesterolemia^(^^10^^)^. In addition, there are no long-term clinical or imaging sequelae of TGA episodes. Furthermore, the risk of stroke and seizures is not significantly increased in patients who have experienced an episode of TGA^(^^1^^)^.

Because many patients have reported that a Valsalva maneuver precipitated a TGA episode, it has been suggested that this maneuver, by preventing venous return via the superior vena cava, could allow a brief retrograde transmission of high venous pressure from the arms to the cerebral venous system, resulting in venous ischemia in the diencephalon or mesiotemporal region of the temporal lobe and, consequently, TGA^(^^12^^)^.

Some studies have shown a strong association between a history of migraine and the risk of TGA, especially in female patients 40-60 years of age^(^^6^^,^^9^^)^. However, few patients have reported active migraine in the months preceding an acute TGA episode or during the episode, suggesting that TGA is not a reflection of an acute migraine^(^^10^^)^.

There are a number of conditions that can mimic the clinical presentation of TGA. The main differential diagnoses (diagnostic approaches) are as follows^(^^3^^)^: transient ischemic attack in the vascular territory of the posterior cerebral artery (assess cardiovascular risk factors); epileptic disorders (perform electroencephalography, especially in cases of brief, recurrent episodes); hypoglycemia, notably in young individuals with diabetes; post-traumatic brain injury, drug intoxication, herpetic encephalitis, or limbic encephalitis (usually in association with focal neurological signs and confusion) and psychiatric disorders.

The rate of detection of TGA-inducing lesions on MRI is directly dependent on the optimization of the image acquisition parameters and on the temporal evolution of the lesions^(^^6^^)^. The reported frequency of such lesions detected on DWI ranges from 0% to 84%, the variation being mainly due to the time since the onset of the pathophysiological process. Therefore, if no lesion is detected in the initial DWI study, especially if it is performed within the first few hours after symptom onset, it is recommended that a follow-up DWI study be performed a few days thereafter^(^^6^^)^.

As for optimization of the MRI parameters, the combined use of a higher b value (> 2000 s/mm^2^) and thinner slices (< 3 mm) is ideal for the detection of TGA^(^^6^^)^. In our study, it was possible to observe the added value of the use of the optimized diffusion sequence, without which the focal lesions would not have been detected in one patient.

Our study has some limitations, not the least of which is its retrospective and essentially descriptive character. In addition, the sample was small. Those factors precluded the in-depth analysis of topics such as intraobserver and interobserver agreement, as well as limiting our ability to make inferences with any level of statistical significance regarding the added value of the optimized diffusion sequence. However, prospective studies involving a larger number of patients could, with a greater degree of statistical precision, establish the role of the optimized diffusion sequence of and the impact that the time to performing the MRI examination after the onset of symptoms has on the positivity of the findings.

## CONCLUSION

MRI plays a useful role in confirming the clinical diagnosis of TGA by detecting foci of restricted diffusion in the hippocampus. To improve the efficiency of the method, the use of an optimized diffusion sequence, ideally carried out within a time window of 48-72 h after the event, is recommended.

## References

[1] Arena JE, Rabinstein AA (2015). Transient global amnesia. Mayo Clin Proc.

[2] Bartsch T, Alfke K, Stingele R (2006). Selective affection of hippocampal CA-1 neurons in patients with
transient global amnesia without long-term sequelae. Brain.

[3] Bartsch T, Deuschl G (2010). Transient global amnesia: functional anatomy and clinical
implications. Lancet Neurol.

[4] Quinette P, Constans JM, Hainselin M (2015). Hippocampal modifications in transient global
amnesia. Rev Neurol (Paris).

[5] Sedlaczek O, Hirsch JG, Grips E (2004). Detection of delayed focal MR changes in the lateral hippocampus
in transient global amnesia. Neurology.

[6] Weon YC, Kim JH, Lee JS (2008). Optimal diffusion-weighted imaging protocol for lesion detection
in transient global amnesia. AJNR Am J Neuroradiol.

[7] Caplan L, Vinken PJ, Gruyn GW, Klawans HL (1985). Transient global amnesia. Handbook of clinical neurology.

[8] Hodges JR, Warlow CP Syndromes of transient amnesia: towards a classification. A study
of 153 cases. J Neurol Neurosurg Psychiatry.

[9] Lin KH, Chen YT, Fuh JL (2014). Migraine is associated with a higher risk of transient global
amnesia: a nationwide cohort study. Eur J Neurol.

[10] Quinette P, Guillery-Girard B, Dayan J (2006). What does transient global amnesia really mean? Review of the
literature and thorough study of 142 cases. Brain.

[11] Hodges JR, Warlow CP (1990). Syndromes of transient amnesia: towards a classification. A study
of 153 cases. J Neurol Neurosurg Psychiatry.

[12] Lewis SL (1998). Aetiology of transient global amnesia. Lancet.

